# Supplementary material for: Differential Gene Expression of Subcutaneous Adipose Tissue among Lean, Obese, and after RYGB (Different Timepoints): Systematic Review and Analysis
Source: Nutrients. 2022 Nov 21;14(22):4925. doi: 10.3390/nu14224925 (PMC9693162; doi:10.3390/nu14224925)
Supplement: Supplementary file 1 [file nutrients-14-04925-s001.zip › Table S1. Characteristics of all the studies included in this systematic review revfin 091122.pdf]

Table S1. Characteristics of all the studies included in this systematic review.

Post-RYGB vs obese

Method	Characteristics of the groups	Bariatric surgery data	BMI data (kg/m^2)	Gene expression fold change (Post-RYGB vs obese): Reported gene expression values (Obese; 8±5 months post-RYGB)	Gene expression fold change (Post-RYGB vs obese): Calculated fold change [(Post-RYGB/Obese)-1]	Reference
	Patients data/Recruitment country			**Upregulated expression post-RYGB(+)**		
**qPCR**<br><br>Candidate genes involved in:<br><br>(a) Lipolysis<br>(b) Triglyceride metabolism	**Obese:**<br>19 obese of ≥ 18 years<br><br>**Post-RYGB:**<br>19 patients post-RYGB of ≥ 18 years<br><br>Boston, United States of America	RYGB<br><br>8±5 months<br><br>(3 a 15 months)	**Obese:**<br>42± 5<br><br>**Post-RYGB:**<br>~32 ± 6<br><br>**ΔBMI:**<br>~10 kg/m^2	***PLIN1* (1.00; 4.01)**<br>***HSL* (1.00; 2.19)**<br>***CG158* (1.00; 2.19)**<br>***ATGL* (1.00; 1.72)**<br><br>Note: Approximate data of expression of the article	**Upregulated expression post-RYGB(+)**<br><br>***PLIN1* (3.01)**<br>***HSL* (1.19)**<br>***CG158* (1.19)**<br>***ATGL* (0.72)**	(Karki et al., 2015)

Post-RYGB vs obese

Method	Characteristics of the groups	Bariatric surgery data	BMI data (kg/m^2)	Gene expression fold change (Post-RYGB vs obese): Calculated fold change [(Post-RYGB/Obese)-1] — Upregulated expression post-RYGB(+)	Gene expression fold change (Post-RYGB vs obese): Calculated fold change [(Post-RYGB/Obese)-1] — Downregulated expression post-RYGB(-)-	Reference
	Patients data/ Recruitment country					
**qPCR**<br><br>Genes involved in:<br><br>(a) Inflammation<br>(b) Lipid metabolism<br>(c) Glucose metabolism	**Obese:**<br>17 Obese of 46 ± 10 years<br><br>**Post-RYGB:**<br>17 patients post-RYGB of 46 ± 10 years<br><br>Barcelona, Spain	RYGB<br><br>90-120 min	**Obese:**<br>~45 ± 3<br><br>**Post-RYGB:**<br>Not changes<br><br>**ΔBMI:**<br>Not changes	***IL-6* (38.37)**<br>***IL-8* (19.75)**<br>*FASN* (1.69)<br>***AQP9* (1.45)**<br>***TNFα* (0.86)**<br>***LBP* (0.72)**<br>***SREBF1* (0.27)**<br>*PPARG* (0.20)<br>*SLC27A2* (0.07)<br>*ACLY* (0.06)<br>*ELOVL6* (0.05)<br>*LEP* (0.02)	*ADIPOQ* (-0.38)<br>***CEBPα* (-0.28)**<br>***IRS-1* (-0.23)**<br>*GLUT4* (-0.23)<br>*ACACA* (-0.09)	(Ortega et al., 2016)

Genes, method, N, mean age, BMI (body mass index), reported or calculated fold change, upregulated or downregulated genes, reference. Highlighted genes are those found in the intersection of comparisons between post-RYGB vs obese and lean vs obese. Bold font appoints significance.

Post-RYGB vs obese

Method	Characteristics of the groups	Bariatric surgery data	BMI data (kg/m^2)	Gene expression fold change (Post-RYGB vs obese)				Reference
	Patients data/Recruitment country			Reported gene expression values (Obese; 6 months post-RYGB)		Calculated fold change [(Post-RYGB/Obese)-1]		
				Upregulated expression post-RYGB(+)	Downregulated expression post-RYGB (-)	Upregulated expression post-RYGB(+)	Downregulated expression post-RYGB (-)	
**qPCR**<br><br>Validation by qPCR of genes involved in:<br><br>(a) Energy metabolism	**Obese:**<br>13 Obese of ~33±9 years<br><br>**Post-RYGB:**<br>13 patients post-RYGB ~33±9 years<br><br>Sao Paulo, Brazil	RYGB<br><br>6 months	**Obese:**<br>~45± 6<br><br>**Post-RYGB:**<br>~35 ± 6<br><br>**ΔBMI:**<br>~10 kg/m^2	***UCP2*** **(0.75; 1.27)**<br><br>Note: Approximate values from the graphs in the article	*PLIN1* (1.16; 0.89)<br><br>Note: Approximate values from the graphs in the article	***UCP2*** **(0.69)**	*PLIN1* (-0.23)<br><br>Note: Approximate values from the graphs in the article	(de Oliveira et al., 2017)

Post-RYGB vs obese

Method	Characteristics of the groups	Bariatric surgery data	BMI data (kg/m^2)	Gene expression fold change (Post-RYGB vs obese)			Reference
	Patients data/Recruitment country			Calculated fold change [(Post-RYGB/Obese)-1]			
				Upregulated expression post-RYGB (+)		Downregulated expression post-RYGB(-)	
**qPCR**<br><br>Validation by qPCR of genes involved in:<br><br>(a) Adipogenesis<br>(b) Oxidation of fatty acids<br>(c) Glucose metabolism	**Obese:**<br>11 Obese of ~55±9 years<br><br>**Post-RYGB:**<br>11 patients post-RYGB ~55±9 years<br><br>Uppsala, Sweden	RYGB<br><br>1 month	**Obese:**<br>~37± 4<br><br>**Post-RYGB:**<br>~33 ± 3<br><br>**ΔBMI:**<br>~4 kg/m^2	*ADIPOQ (3.81)*<br>*PLIN1* (2.84)<br>***FABP4*** **(1.7)**<br>*LIPE* (1.12)<br>*FAS* (0.76)<br>*CD36* (0.68)<br>***CPT1B*** **(0.53)**<br>*SLC2A4* (0.32)<br>*IL-6* (0.23)<br>*PPARG* (0.22)<br>*CEBPA* (0.06)	*ACACA* (0.22)<br>*ACLY* (0.22)<br>*AKT1* (0.21)<br>*IRS-1* (0.2)<br>*CPT1A* (0.16)<br>*SLC2A1* (0.15)<br>*CASP3* (0.15)<br>*LPL* (0.09)<br>*PNPLA2* (0.07)	*IL-18* (-0.34)<br>*MKI67* (-0.30)<br>***E2F1*** **(-0.29)**<br>***LEP*** **(-0.25)**<br>***ELOVL6*** **(-0.19)**<br>*SREBF1* (-0.19)<br>*TNFα* (-0.15)	(Katsogiannos et al., 2019)

| | | | | *CEBPB* (0.24) | | | |

Genes, method, N, mean age, BMI (body mass index), reported or calculated fold change, upregulated or downregulated genes, reference. Highlighted genes are those found in the intersection of comparisons between post-RYGB vs obese and lean vs obese. Bold font appoints significance.

Post-RYGB vs obese

Method	Characteristics of the groups	Bariatric surgery data	BMI data (kg/m^2)	Gene expression fold change (Post-RYGB vs obese)		Reference
	Patients data/Recruitment country			Calculated fold change [(Post-RYGB/Obese)-1]		
				**Upregulated expression post-RYGB(+)**	**Downregulated expression post-RYGB(-)**	
**qPCR**<br><br>Candidate genes involved in:<br>a) Adipogenesis<br>b) Oxidation of fatty acids<br>c) Glucose metabolism	**Obese:**<br>12 obese of 55 ± 9 years<br><br>**Post-RYGB:**<br>12 patients post-RYGB of 55 ± 9 years<br><br>Uppsala, Sweden	RYGB<br><br>6 months	**Obese:**<br>37± 4<br><br>**Post-RYGB:**<br>~29 ± 3<br><br>**ΔBMI:**<br>~8 kg/m^2;	***ADIPOQ (11.15)***<br>***FAS (4.67)***<br>***SLC2A4 (2.57)***<br>***FABP4 (1.91)***<br>***PPARG (1.14)***<br>***CPT1B (1.13)***<br>*CEBPA (0.88)*<br>*LIPE (0.86)*<br>*ELOVL6 (0.68)*<br>***IRS-1 (0.66)***<br>***CEBPB (0.64)***<br>***AKT1 (0.37)***<br>*SLC2A1 (0.28)*<br>*LEP (0.19)*	***E2F1 (-0.33)***	(Katsogiannos et al., 2019)
				Note: Upregulated expression involved in adipogenesis	Note: Downregulated genes involved in cell proliferation	

Post-RYGB vs obese

Method	Characteristics of the groups	Bariatric surgery data	Patients data	Gene expression fold change (Post-RYGB vs obese)				Reference
	Patients data/Recruitment country			Reported gene expression values **(Obese; 3 months post-RYGB)**		**Calculated fold change [(Post-RYGB/Obese)-1]**		
				**Upregulated expression post-RYGB(+)**	**Downregulated expression post-RYGB (-)**			
				Logarithmic conversion	Logarithmic conversion	**Upregulated expression post-RYGB(+)**	**Downregulated expression post-RYGB (-)**	
**qPCR**<br><br>Validation by qPCR of genes involved in<br>(a) Inflammatory and anti-inflammatory cytokines<br>(b) Lipid transport<br>(c) Metabolic regulation<br>(d) In caloric restriction<br>(e)Glucose homeostasis<br>(f) Oxidative stress	**Obese:**<br>13 Obese of ~38±8 years<br><br>**Post-RYGB:**<br>13 patients of ~38±8 years<br><br>Sao Paulo, Brazil	RYGB<br><br>3 months	**Obese:**<br>~42± 4<br><br>**Post-RYGB:**<br>~36 ± 5<br><br>**ΔBMI:**<br>~6 kg/m^2	***NRF2 (0.84; 4.50)***<br>***AMPK (0.92; 4.14)***<br>***ADIPOQ (0.80; 3.29)***<br>***SIRT1 (0.84; 3.29)***<br>***GNC2 (0.97; 3.07)***<br>***ATF4 (0.82; 2.55)***<br>***PGC1α (0.86; 2.28)***<br>***SIRT3 (0.81; 1.88)***<br>***SOD2 (0.93; 1.53)***<br>*ATF6 (0.6; 0.96)*<br>*CANX (0.59; 0.75)*<br>*SOD3 (0.78; 0.91)*<br>*CHOP (0.61; 0.71)*<br>*XBP1 (0.91; 0.97)*<br>*SOD1 (0.91; 0.95)*<br>Note: Approximate values from the graphs in the article	***CALR (0.82; 0.05)***<br>***EIF2AK3 (0.85; 0.15)***<br>***MCP-1 (0.89; 0.25)***<br>*TNFα (0.79; 0.28)*<br>***IL-6 (0.86; 0.37)***<br>***PPARG (0.97; 0.45)***<br>*GRP78 (0.45; 0.25)*<br>*CCT4 (0.91; 0.8)*<br>*GADD34 (0.88; 0.84)*<br><br>Note: Approximate values from the graphs in the article	***NRF2 (4.35)***<br>***AMPK (3.5)***<br>***ADIPOQ (3.11)***<br>*SIRT1 (2.94)*<br>*GNC2 (2.16)*<br>*ATF4 (2.12)*<br>***PGC1α (1.64)***<br>***SIRT3 (1.33)***<br>***SOD2 (0.65)***<br>*ATF6 (0.59)*<br>*CANX (0.27)*<br>*SOD3 (0.16)*<br>*CHOP (0.16)*<br>*XBP1 (0.06)*<br>*SOD1 (0.04)*	*CALR (-0.94)*<br>*EIF2AK3 (-0.83)*<br>*MCP-1 (-0.72)*<br>*TNFα (-0.64)*<br>***IL-6 (-0.57)***<br>***PPARG (-0.53)***<br>*GRP78 (-0.45)*<br>*CCT4 (-0.13)*<br>*GADD34 (-0.04)*	(Ferraz-Bannitz et al., 2021)

Genes, method, N, mean age, BMI (body mass index), reported or calculated fold change, upregulated or downregulated genes, reference. Highlighted genes are those found in the intersection of comparisons between post-RYGB vs obese and lean vs obese. Bold font appoints significance.

Post-RYGB vs obese

Method	Characteristics of the groups	Bariatric surgery data	BMI data (kg/m^2)	Gene expression fold change (Post-RYGB vs obese)				Reference
	Patients data/Recruitment country			Reported gene expression values **(Obese; 6 months post-RYGB)**		**Calculated fold change [(Post-RYGB/Obese)-1]**		
				**Upregulated expression post-RYGB(+)**	**Downregulated expression post-RYGB (-)**			
				Logarithmic conversion	Logarithmic conversion	**Upregulated expression post-RYGB(+)**	**Downregulated expression post-RYGB (-)**	
**qPCR**<br>**(a)** Validation by qPCR of genes involved in:<br>**(b)** Inflammatory and anti-inflammatory cytokines<br>**(c)** Lipid transport<br>**(d)** Adipogenesis<br>**(e)** Metabolic regulation<br>**(f)** Amino acid metabolism<br>**(g)** In caloric restriction<br>**(h)** Glucose homeostasis<br>**(i)** Oxidative stress	**Obese:**<br>13 Obese of ~38±8 years<br>**Post-RYGB:**<br>13 patients of ~38±8 years<br>Sao Paulo, Brazil	RYGB<br>6 months	**Obese:**<br>~42± 4<br>**Post-RYGB:**<br>~33 ± 3<br>**ΔBMI:**<br>~9 kg/m^2;	***ADIPOQ (0.80; 4.89)***<br>***SIRT1 (0.84; 2.85)***<br>***NRF2 (0.84; 2.75)***<br>***AMPK (0.92; 2.68)***<br>***GNC2 (0.97; 2.5)***<br>***ATF4 (0.82; 2.08)***<br>***PGC1α (0.86; 1.77)***<br>***SIRT3 (0.81; 1.59)***<br>*ATF6 (0.60; 1.19)*<br>*GRP78 (0.45; 0.89)*<br>*CHOP (0.61; 1.14)*<br>*CANX (0.59; 0.82)*<br>*SOD2 (0.93; 1.15)*<br>*XBP1 (0.91; 1.05)*<br>*SOD3 (0.78; 0.81*<br><br>Note: Approximate values from article graphics	***CALR (0.82; 0.14)***<br>***EIF2AK3 (0.85; 0.15)***<br>***TNFα (0.79; 0.17)***<br>***PPARG (0.97; 0.61)***<br>*GADD34 (0.88; 0.59)*<br>*IL-6 (0.86; 0.66)*<br>*SOD1 (0.91; 0.8)*<br>*CCT4 (0.91; 0.82)*<br>*MCP-1 (0.89; 0.86)*<br><br>Note: Approximate values from article graphics	***ADIPOQ (5.11)***<br>***SIRT1 (2.41)***<br>***NRF2 (2.27)***<br>***AMPK (1.91)***<br>***GNC2 (1.57)***<br>***ATF4 (1.55)***<br>***PGC1α (1.04)***<br>***SIRT3 (0.97)***<br>*ATF6 (0.97)*<br>*GRP78 (0.97)*<br>*CHOP (0.87)*<br>*CANX (0.39)*<br>*SOD2 (0.24)*<br>*XBP1 (0.15)*<br>*SOD3 (0.04*	***CALR (-0.83)***<br>***EIF2AK3 (-0.83)***<br>***TNFα (-0.79)***<br>***PPARG (-0.37)***<br>*GADD34 (-0.33)*<br>*IL-6 (-0.23)*<br>*SOD1 (-0.12)*<br>*CCT4 (-0.1)*<br>*MCP-1 (-0.03)*	(Ferraz-Bannitz, et al., 2021)

Genes, method, N, mean age, BMI (body mass index), reported or calculated fold change, upregulated or downregulated genes, reference. Highlighted genes are those found in the intersection of comparisons between post-RYGB vs obese and lean vs obese. Bold font appoints significance.

Post-RYGB vs obese

Method	Characteristics of the groups	Bariatric surgery data	BMI data (kg/m^2)	Gene expression fold change (Post RYGB vs Obese)				Reference
	Patients data/Recruitment country			Microarrays		qPCR		
				Calculated fold change [(Post-RYGB/Obese)-1]				
				**Downregulated expression post-RYGB(+)**	**Downregulated expression post-RYGB(-)**	**Downregulated expression post-RYGB(+)**	**Downregulated expression post-RYGB(-)**	
. **Microarrays**<br><br>SurePrint-G3 Human GE 8 x60 K microarray kit 62,000 transcripts<br><br>**qPCR**<br><br>Validation by qPCR of genes selected for antecedents of improving lipid metabolism and post-RYGB inflammation<br><br>In addition, those that were differentially expressed in Microarrays were selected.	**Microarrays**<br><br>**Obese:**<br>8 Obese of ~41±11 years<br><br>**Post-RYGB:**<br>8 patients<br><br>**qPCR**<br><br>**Obese:**<br>20 obese of ~46 ± 10 years<br><br>**Post-RYGB:**<br>20 patients<br><br>Malaga, Spain	RYGB<br><br>~2 years	**Microarrays**<br><br>**Obese:**<br>~52± 7<br><br>**Post-RYGB:**<br>~34 ± 9<br><br>**ΔBMI:**<br>~18 kg/m^2;<br><br>**qPCR**<br><br>**Obese:**<br>~56 ± 7<br><br>**Post-RYGB:**<br>~39 ± 10<br><br>**ΔBMI:**<br>~17 kg/m^2;	*172 upregulated genes post-RYGB*<br><br>Validation genes:<br>***FASN (4.94)***<br>***ACACA (2.13)***<br>***SCD1 (2.13)***<br>***IRS-1 (1.19)***<br><br>Note: Upregulated expression implied in:<br>(a) Biosynthetic processes<br><br>Note: Approximate values from the graphs in the article	*731 downregulated genes post-RYGB*<br><br>Validation genes:<br>***IL-6*** **(-57.67)**<br>***MSR1*** **(-10.69)**<br>***TNFα (-4.75)***<br>***FBP1 (-5.06)***<br><br>Note: Downregulated genes involved in:<br><br>(a) Immune system<br>(b) Inflammatory processes (immune response, stress response, cell death, response to endogenous stimulus)<br><br>Note: Approximate values from the graphs in the article	***FASN (5.88)***<br>***ACACA (2.13)***<br>***SCD1 (2.13)***<br>***IRS-1 (1.5)***<br><br>Note: Approximate values from the graphs in the article	***IL-6*** **(-7.56)**<br>***MSR1*** **(-7.56)**<br>***TNFα (-2.88)***<br>***FBP1*** **(-3.50)**<br><br>Note: Approximate values from the graphs in the article	(González-Plaza et al., 2016)

Genes, method, N, mean age, BMI (body mass index), reported or calculated fold change, upregulated or downregulated genes, reference. Highlighted genes are those found in the intersection of comparisons between post-RYGB vs obese and lean vs obese. Bold font appoints significance.

Post-RYGB vs obese

Method	Characteristics of the groups	Bariatric surgery data	BMI data (kg/m²)	Gene expression fold change (Post RYGB vs Obese)				Reference
	Patients data/Recruitment country			Microarrays		qPCR		
				Calculated fold change [(Post-RYGB/Obese)-1]				
				**Upregulated expression post-RYGB(+)**	**Downregulated expression post-RYGB(-)**	**Upregulated expression post-RYGB(+)**	**Downregulated expression post-RYGB(-)**	
**Microarrays**<br><br>SurePrint-G3 Human GE 8 x60 K microarray kit >55,077 transcripts<br><br>Focus on genes involved in a state of low and high insulin resistance (IR), for a possible relationship with comorbidities.<br><br>**qPCR**<br><br>Subsequent validation of genes identified.	**Microarrays**<br><br>**OBESE:**<br>8 Obese<br>[4 down-insulin resistance (low-IR);<br>4 high-insulin resistance (high-IR)]<br><br>***Low-IR:***~39±13 years<br>***High-IR:***~43±11 years<br><br>**qPCR**<br><br>**OBESE:**<br><br>9 obese<br>(9 low-IR; 11 high-IR:)<br><br>***Low-IR:***~45±13 years<br>***High-IR:***~47±9 years<br><br>Malaga, Spain	RYGB<br><br>~2 years	**Microarrays**<br><br>**OBESE**<br><br>***Low-IR:*** ~49±5<br>***High-IR:***~52±7<br><br>**POST-RYGB:**<br><br>***Low-IR:*** ~32±6<br>***High-IR:***~37±3<br><br>**ΔBMI:**<br><br>***Low-IR:***~17 kg/m²<br>***High-IR:***~16 kg/m²<br><br>**qPCR**<br><br>**OBESE**<br><br>***Low-IR:***~57±9<br>***High-IR:***~55±6<br><br>**POST-RYGB:**<br><br>***Low-IR:***~39±9<br>***High-IR:***~38±6<br><br>**ΔBMI:**<br><br>***Low-IR:***~18 kg/m²<br>***High-IR:***~17 kg/m²	*Logarithmic conversion*<br><br>*153 upregulated genes post-RYGB:*<br><br>**High-IR:**<br><br>*GLYCTK (2.18)*<br>***IRS-1* (1.27)**<br>*FADS1 (0.55)*<br><br>*227 upregulated genes post-RYGB:*<br><br>**Low-IR:**<br><br>***GLYCTK* (4.73)**<br>***FADS1* (2.55)**<br>***IRS-1* (1.27)**<br><br>Note: In low-IR, genes involved in carbohydrate metabolic processes<br><br>Note: Approximate values from the graphs in the article	*Logarithmic conversion*<br><br>*865 downregulated genes post-RYGB:*<br><br>**High-IR:**<br><br>***ITPKC*(-7.18)**<br>***TNFα* (-5.82)**<br>***LIF* (-4.73).**<br>*UCHL1 (-4.36)*<br>*CCND1 (-2.36)*<br><br>*619 downregulated genes post-RYGB:*<br><br>**Low-IR:**<br><br>***UCHL1* (-8.55)**<br>***CCND1* (-5.09)**<br>***TNFα* (-4.64)**<br>*ITPKC (-2.91)*<br>*LIF (-2.91)*<br><br>Note: In high-IR genes involved in cytokine-cytokine receptor, cancer chemokine signaling, and activation of transcription factors<br><br>In low-IR, genes involved in glycosaminoglycan metabolism and translation regulation	**High-IR:**<br><br>***IRS-1* (2.73)**<br>***FADS1* (1.64)**<br>*GLYCTK (3.09)*<br><br>**Low-IR:**<br><br>***GLYCTK* (4.55)**<br>***FADS1* (3.45)**<br>***IRS-1* (2.36)**<br><br>Note: Approximate values from the graphs in the article	**High-IR:**<br><br>***ITPKC* (-5.27)**<br>***LIF* (-4.73)**<br>***TNFα*(-3.27)**<br>*UCHL1 (-4.10)*<br>*CCND1 (-2.55)*<br><br>**Low-IR:**<br><br>***UCHL1*(-6.00)**<br>***CCND1*(-4.10)**<br>***TNFα*(-2.91)**<br>*ITPKC (-5.27)*<br>*LIF (-2.91)*<br><br>Note: Approximate values from the graphs in the article	(González-Plaza et al., 2018)

Genes, method, N, mean age, BMI (body mass index), reported or calculated fold change, upregulated or downregulated genes, reference. Highlighted genes are those found in the intersection of comparisons between post-RYGB vs obese and lean vs obese. Bold font appoints significance.

Post-RYGB vs obese

Method	Characteristics of the groups	Bariatric surgery data	BMI data (kg/m^2)	Gene expression fold change (Post RYGB vs Obese): Microarrays – Calculated fold change [(PostRYGB/ Obese)-1]	qPCR – Reported fold change values (Obese; 2 years post-RYGB): Upregulated expression post-RYGB(+)	qPCR – Reported fold change values (Obese; 2 years post-RYGB): Downregulated expression post-RYGB(-)	qPCR – Calculated fold change [(Post-RYGB/Obese)-1]: Upregulated expression post-RYGB(+)	qPCR – Calculated fold change [(Post-RYGB/Obese)-1]: Downregulated expression post-RYGB(-)	Reference
	Patients data/Recruitment country			Logarithmic conversion					
<u>**Microarrays**</u><br><br>Affymetrix GeneChip Human Gene 2.0 ST Array<br>13,885 transcripts post-RYGB VS obese<br>(Ortega et al., 2015)<br><br><u>**qPCR**</u><br><br>Candidate genes involved in:<br>(a) Inflammatory<br>(b) Toll-like receptors (TLRs)	<u>**Microarrays**</u><br><br>**Obese:**<br>16 obese of 48 ± 10 years<br><br>**Post-RYGB:**<br>16 patients<br><br><u>**qPCR**</u><br><br>**Obese:**<br>22 obese of 49 ± 8 years<br><br>**Post-RYGB:**<br>22 patients of 53 ± 8 years<br><br>Barcelona, Spain	RYGB<br><br>~2 years	<u>**Microarrays**</u><br><br>**Obese:**<br>~46 ± 6<br><br>**Post-RYGB:**<br>Not mentioned<br><br><u>**qPCR**</u><br><br>**Obese:**<br>~43 ± 5<br><br>**Post-RYGB:**<br>~30 ± 5<br><br>**ΔBMI:**<br>~13 kg/m^2	**Downregulated expression post-RYGB(+)**<br><br>***TLR8 (-0.53)***<br>***TLR7 (-0.48)***<br>***TLR1 (-0.44)***<br>***TLR2 (-0.36)***<br>***TLR5 (-0.35)***<br>***TLR6 (-0.34)***<br>***TLR4 (-0.13)***<br>***TLR3 (-0.12)***<br>*TLR10 (-0.05)*<br>*TLR9 (-0.05)*<br><br>Note: Microarray results from Ortega et al., 2015	***GLUT4 (0.04472;0.08758)***<br><br>***ADIPOQ (3.24; 4.57)***<br><br>***IRS-1 (0.0107;0.01438)***	***IL-6 (0.01695; 0.0006)***<br><br>***TNFα (0.00302; 0.00074)***<br><br>***TLR8 (0.00032; 0.00011)***<br><br>***LEP (0.9706; 0.3327)***<br><br>***TLR1 (0.01177;0.00582)***<br><br>*TLR7 (0.00737;0.00466)*<br><br>*TLR4 (0.02932; 0.0196)*<br><br>*TLR5 (0.00398;0.00269)*<br><br>*TLR2 (0.00821;0.00575)*<br><br>*TLR6 (0.00666;0.00482)*<br><br>***TLR3 (0.01014;0.00828)***	***GLUT4 (0.96)***<br>***ADIPOQ (0.41)***<br>***IRS-1 (0.35)***	***IL-6 (-0.96)***<br><br>***TNFα (-0.75)***<br><br>***TLR8 (-0.66)***<br><br>***LEP (-0.66)***<br><br>***TLR1 (-0.51)***<br><br>*TLR7 (-0.37)*<br><br>*TLR4 (-0.33)*<br><br>*TLR5 (-0.32)*<br><br>*TLR2 (-0.3)*<br><br>*TLR6 (-0.28)*<br><br>***TLR3 (-0.18)***	(Latorre et al., 2018)

Genes, method, N, mean age, BMI (body mass index), reported or calculated fold change, upregulated or downregulated genes, reference. Highlighted genes are those found in the intersection of comparisons between post-RYGB vs obese and lean vs obese. Bold font appoints significance.

Post-RYGB vs obese

Method	Characteristics of the groups	Bariatric surgery data	BMI data (kg/m²)	Gene expression fold change (Post-RYGB vs obese)			Reference
	Patients data/Recruitment country			Calculated fold change [(Post-RYGB/Obese)-1]			
				Upregulated expression post-RYGB (+)	Downregulated expression post-RYGB (-)		
. <u>**Microarrays**</u><br><br>Human HT12 v4.0 BeadChip 47,231 transcripts<br><br>Selection of genes involved in:<br>• Matrix fibers<br>• Cross-linking enzymes<br>• Profibrotic proteins<br>• Glycosaminoglycan proteins<br>• Multitensive glycoproteins<br>• MMPs/TIMPs<br>Post-transcriptional modification of collagen	<u>**Microarrays**</u><br><br>**Obese:**<br>42 obese of ~43±11 years<br><br>**Post-RYGB:**<br>42 patients<br><br>Paris, France	RYGB<br><br>~1 year	<u>**Microarrays**</u><br><br>**Obese:**<br>~47± 6<br><br>**Post-RYGB:**<br>~33 ± 5<br><br>**ΔBMI:**<br>~15 kg/m²;	*4, 236 upregulated genes post-RYGB*<br><br>***TIMP4 (0.52)***<br>***LAMB1 (0.43)***<br>*COL1A1 (0.36)*<br>***COL6A3 (0.26)***<br>***THBS2 (0.24)***<br>***LAMA2 (0.17)***<br>***P3H2 (0.16)***<br>***ADAMTS9 (0.16)***<br>*TGM2 (0.14)*<br>*FBLN1 (0.14)*<br>*TBHS4 (0.14)*<br>***HSPG2 (0.1)***<br>***LAMA3 (0.1)***<br>*LAMA4 (0.1)*<br>***P3H3 (0.09)***<br>*COL4A2 (0.05)*<br>*VIT (0.05)*<br>*TGM3 (0.03)*<br>*PLOD1 (0.03)*<br>*MMP2 (0.03)*<br>*LAMA5 (0)*<br><br>Note: Approximate values from graphics in the article	*2,989 downregulated genes post-RYGB*<br><br>*MMP9 (-0.62)*<br>*SPP1 (-0.59)*<br>*LOX (-0.41)*<br>*SPARC (-0.36)*<br>*COL6A2 (-0.31)*<br>*CTGF (-0.28)*<br>*TIMP1 (-0.28)*<br>*COL6A1 (-0.240)*<br>*LOXL4 (-0.240)*<br>*HSPA5 (-0.23)*<br>*COL3A1 (-0.2)*<br>*TGM1 (-0.17)*<br>*TNC (-0.17)*<br>*GLT25D1 (-0.16)*<br>*HSP47 (-0.16)*<br>*PLOD2 (-0.14)*<br>*CANX (-0.14)*<br>*ADAMTSL4 (-0.14)*<br>*PDIA6 (-0.13)*<br>*P4HA2 (-0.11)*<br>*P4HB (-0.11)*<br>*BMP1 (-0.11)*	***PLOD3 (-0.1)***<br>***TIMP2 (-0.09)***<br>***ADAMTS1 (-0.09)***<br>***ADAMTS5 (-0.09)***<br>***P4HA2 (-0.09)***<br>***PDIA4 (-0.08)***<br>***ELN (-0.07)***<br>***THBS1 (-0.07)***<br>***DNAJC10 (-0.07)***<br>***P4HA1 (-0.05)***<br>***P3H1 (-0.05)***<br>***HSP90AA1 (-0.05)***<br>***HSP90B1 (-0.05)***<br>***ERP29 (-0.05)***<br>***ADAMTS2 (-0.05)***<br>***COL4A1 (-0.04)***<br>***COL4A5 (-0.04)***<br>*MMP11 (-0.02)*<br>*LOXL1 (-0.02)*<br><br>Note: Downregulated genes encoding cross-linking enzymes<br><br>Note: Approximate values from graphics in the article	(Liu et al., 2016)

Genes, method, N, mean age, BMI (body mass index), reported or calculated fold change, upregulated or downregulated genes, reference. Highlighted genes are those found in the intersection of comparisons between post-RYGB vs obese and lean vs obese. Bold font appoints significance.

Post-RYGB vs obese

Method	Characteristics of the groups	Bariatric surgery data	BMI data (kg/m²)	Gene expression fold change (Post RYGB vs Obese)		Reference
	Patients data/Recruitment country			Reported gene expression values (Obese; 1 week post-RYGB)<br>Upregulated expression post-RYGB (+)	Calculated fold change [(Post-RYGB/Obese)-1]	
**qPCR**<br>Validation by qPCR of genes involved in:<br>(a) Mitochondrial biogenesis<br>(b) Oxidative stress	**Obese:**<br>8 obese of ~50 ± 3 years<br>**Post-RYGB:**<br>8 patients of ~50 ± 3 years<br>Minnesota, United States of America	RYGB<br>1 week	**Obese:**<br>~40 ± 2<br>**Post-RYGB:**<br>~39 ± 2<br>**ΔBMI:**<br>~1 kg/m²	***eNOS*** **(1.00; 3.77)**<br>***NRF1*** **(1.00; 3.64)**<br>***Tfam*** **(1.00; 2.30)**<br>***PGC1α*** **(1.00; 1.47)**<br>***CytC*** **(1.00; 1.74)**<br>Note: Approximate values from article graphics	**Upregulated expression post-RYGB (+)**<br>***eNOS*** **(2.77)**<br>***NRF1*** **(2.64)**<br>***Tfam*** **(1.29)**<br>***PGC1α*** **(0.47)**<br>***CytC*** **(0.74)**	(Jahansouz et al., 2015)

Lean vs obese

Method	Characteristics of the groups		Gene expression fold change (Lean vs Obese)			Reference
	Patients data/Recruitment country	BMI (kg/m²)	Reported gene expression values (Obese; Lean)<br>Upregulated expression in lean	Downregulated genes in lean (-)	Calculated fold change [(Lean/Obese)-1]	
**qPCR**<br>Candidate genes involved in:<br>(a) Mitochondrial biogenesis<br>(b) Oxidative stress	**Lean:**<br>8 lean of 45 ± 6 years<br>**Obese:**<br>16 obese of 46 ± 3 years<br>Minnesota, United States of America	**Lean:**<br>24±0.9 kg/m²<br>**Obese:**<br>41± 1 kg/m²	***CytC (0.040; 1.00)***<br>***eNOS (0.20; 1.00)***<br>***NRF1 (0.260; 1.00)***<br>*TFAM (0.48; 1.00)*<br>***PGC1α (0.60; 1.00)***<br>Note: Approximate values from article graphics	Not reported	**Downregulated genes in lean (-)**<br>***CytC (24.00)***<br>***eNOS (4.00)***<br>***NRF1 (2.85)***<br>*TFAM (1.08)*<br>***PGC1α (0.67)***	(Jahansouz et al., 2015)

Genes, method, N, mean age, BMI (body mass index), reported or calculated fold change, upregulated or downregulated genes, reference. Highlighted genes are those found in the intersection of comparisons between post-RYGB vs obese and lean vs obese. Bold font appoints significance.

Post-RYGB vs obese

<u>Method</u>	Characteristics of the groups	Bariatric surgery data	BMI data (kg/m^2)	Gene expression fold change (Post-RYGB vs Obese)				Reference
	Patients data/Recruitment country			Reported gene expression values **(Obese; 1 year post-RYGB)**		**Calculated fold change [(Post-RYGB/Obese)-1]**		
				**Upregulated expression post-RYGB (+)**	**Downregulated expression post-RYGB (-)**	**Upregulated expression post-RYGB(+)**	**Downregulated expression post-RYGB(-)**	
**<u>qPCR</u>**<br><br>Candidate genes involved in:<br><br>(a) Inflammatory cytokines<br>(b) Growth factors<br>(c) Metabolic markers (lipolysis)<br>(d) Cell surface markers (apoptosis**)**	**Obese:**<br>26 obese of ~42±12 years<br><br>**Post-RYGB:**<br>26 patients of ~43±12 years<br><br>Vienna, Austria	RYGB<br><br>~1 year	**Obese:**<br>~46± 6<br><br>**Post-RYGB:**<br>~31 ± 6<br><br>**ΔBMI:**<br>~15 kg/m^2;	***TNFα (1.00; 2.72)***<br>***CASP3 (1.00; 1.64)***<br>*CD40 (0.97; 1.02)*<br><br>Note: Values calculated from the graphs of the article (without %)	***IL-6 (1.00; 0)***<br>***FGF1 (1.00; 0.01)***<br>***IRS-2 (1.00; 0.07)***<br>***CCL3 (1.00; 0.09)***<br>***IL-10 (1.02; 0.10)***<br>***PPARG (1.01; 0.11)***<br>***ADIPOQ (1.00; 0.12)***<br>***IL1B (1.00; 0.12)***<br>***CD68 (0.97; 0.13)***<br>***CIDEA (1.00; 0.13)***<br>***PLIN1 (1.00; 0.13)***<br>***IGF1 (1.00; 0.19)***<br>***CD144 (0.92; 0.25)***<br>***SLC2A4 (1.01; 0.31)***<br>***HLA-DR (0.92; 0.40)***<br>***CD3E (0.94; 0.42)***<br>***VEGFC (1.00; 0.96)***<br><br>Note: Of the genes downregulated post-RYGB, 4 are metabolic markers.<br><br>Note: Values calculated from the graphs of the article (without %)	***TNFα (1.72)***<br>***CASP3 (0.64)***<br>*CD40 (0.05)*	***IL-6 (-1.00)***<br>***FGF1 (-0.99)***<br>***IRS-2 (-0.93)***<br>***CCL3 (-0.91)***<br>***IL-10 (-0.9)***<br>***PPARG (-0.89)***<br>***ADIPOQ (-0.88)***<br>***IL1B (-0.88)***<br>***CD68 (-0.87)***<br>***CIDEA (-0.87)***<br>***PLIN1 (-0.87)***<br>***IGF1 (-0.81)***<br>***CD144 (-0.73)***<br>***SLC2A4 (-0.69)***<br>***HLA-DR (-0.57)***<br>***CD3E (-0.55)***<br>***VEGFC (-0.04)***	(Jürets et al., 2017)

Genes, method, N, mean age, BMI (body mass index), reported or calculated fold change, upregulated or downregulated genes, reference. Highlighted genes are those found in the intersection of comparisons between post-RYGB vs obese and lean vs obese. Bold font appoints significance.

Lean vs obese

Method	Characteristics of the groups		Gene expression fold change (Lean vs Obese)				Reference
	Patients data/Recruitment country	BMI (kg/m²)	Reported gene expression values **(Obese; Lean)**		**Calculated fold change [(Lean/Obese)-1]**		
			**Upregulated expression in lean (+)**	**Downregulated expression in lean (-)**	**Upregulated expression in lean(+)**	**Downregulated expression in lean (-)**	
**qPCR**<br><br>Candidate genes involved in:<br><br>(a) Inflammatory cytokines<br>(b) Growth factors<br>(c) Metabolic markers (lipids and glucose)<br>(d) Cell surface markers (apoptosis)	**qPCR**<br><br>**Lean:**<br>20 lean of 43 ± 9 years<br><br>**Obese:**<br>26 obese of 42 ± 12 years<br><br>Vienna, Austria	**qPCR**<br><br>**Lean:**<br>~25 ± 3<br><br>**Obese:**<br>~46 ± 6	*CIDEA (1.00; 1.52)*<br>*CD40 (1.00; 1.17)*<br>*ADIPOQ (1.00; 1.13)*<br>*SLC2A4 (1.00; 1.10)*<br>*IGF1 (1.00; 1.08)*<br>*IRS-2 (1.00; 1.08)*<br>*PLIN1 (1.00; 1.04)*<br>*CD3E (1.00; 1.03)*<br><br>Note: Approximate values from graphics in the article	***IL-6 (1.00; 0.17)***<br>***IL-10 (1.00; 0.37)***<br>***FGF1 (1.00; 0.42)***<br>*CCL3 (1.00; 0.5)*<br>***IL1B (1.00; 0.50)***<br>***CASP3 (1.00; 0.66)***<br>*CD68 (1.00; 0.70)*<br>*HLA-DR (1.00; 0.75)*<br>*CD144 (1.00; 0.75)*<br>*VEGFC (1.00; 0.75)*<br>*PPARG (1.00; 0.88)*<br>*TNFα (1.00; 0.92)*<br><br>Note: Approximate values from graphics in the article	*CIDEA (0.52)*<br>*CD40 (0.17)*<br>*ADIPOQ (0.13)*<br>*SLC2A4 (0.10)*<br>*IGF1 (0.08)*<br>*IRS-2 (0.08)*<br>*PLIN1 (0.04)*<br>*CD3E (0.03)*	***IL-6 (-0.83)***<br>***IL-10 (-0.63)***<br>***FGF1 (-0.58)***<br>*CCL3 (-0.50)*<br>***IL1B (-0.50)***<br>***CASP3 (-0.34)***<br>*CD68 (-0.30)*<br>*HLA-DR (-0.25)*<br>*CD144 (-0.25)*<br>*VEGFC (-0.25)*<br>*PPARG (-0.12)*<br>*TNFα (-0.08)*	(Jürets et al., 2017)

Genes, method, N, mean age, BMI (body mass index), reported or calculated fold change, upregulated or downregulated genes, reference. Highlighted genes are those found in the intersection of comparisons between post-RYGB vs obese and lean vs obese. Bold font appoints significance.

Post-RYGB vs obese

Method	Characteristics of the groups	Bariatric surgery data	BMI data (kg/m^2)	Gene expression fold change (Post RYGB vs Obese)				Reference
	Patients data/Recruitment country					Calculated fold change [(Post-RYGB/Obese)-1]		
				Upregulated expression post-RYGB(+)	Downregulated expression post-RYGB(-)	Upregulated expression post-RYGB(+)	Downregulated expression post-RYGB(-)	
**Microarrays**<br><br>Affymetrix GeneChip Human Gene 2.0 ST Array<br>13,885 transcripts post-RYGB vs obese<br><br>**qPCR**<br><br>Candidate genes involved in:<br><br>(a) Cell cycle<br>(b) Development<br>(c) Lipid metabolism<br>(d) Inflammatory response<br><br>In addition, the genes that were most regulated post-RYGB were selected**.**	**Microarrays**<br><br>**Obese:**<br>16 obese of 48±10 years<br><br>**Post-RYGB:**<br>16 patients of 51 ± 9 years<br><br>**qPCR**<br><br>**Obese:**<br>25 obese of 48±10 years<br><br>**Post-RYGB:**<br>25 patients of 51 ± 9 years<br><br>Girona, Spain	RYGB<br><br>~2 years	**Microarrays and qPCR**<br><br>**Obese:**<br>~43 ± 5<br><br>**Post-RYGB:**<br>~29 ± 6<br><br>**ΔBMI**<br>~14 kg/m^2;	Logarithmic conversion<br><br>*2,432 upregulated genes*<br><br>***SLC27A2 (3.79)***<br>***ELOVL6 (1.91)***<br>***FASN (1.87)***<br>***STOX1 (1.77)***<br>***GYS2 (1.71)***<br>***MOGAT1 (1.58)***<br>***SNORA71B (1.58)***<br>***LGALS12 (1.46)***<br>***C6 (1.45)***<br>***TF (1.45)***<br>***PKP2 (1.43)***<br>***OLFM2 (1.35)***<br>***ACLY (1.35)***<br><br>Note:	Logarithmic conversion<br><br>*2,586 downregulated genes*<br><br>***FOS (-0.86)***<br>***EGFL6 (-0.79)***<br>***AQP9 (-0.78)***<br>***PRG4 (-0.78)***<br>***FOSB (-0.73)***<br>***MT1A (-0.71)***<br>***DUSP1 (-0.7)***<br>***RGS1 (-0.69)***<br>***EGR1 (-0.68)***<br>***SPP1 (-0.67)***<br>***LYZ (-0.67)***<br>***C5AR1 (-0.66)***<br>***IFI30 (-0.66)***<br>***HSD11B1 (-0.65)***<br>***CD69 (-0.63)***<br>***IL8 (-0.63)***<br>***CLEC7A (-0.63)***<br>***JUNB (-0.62)***<br>***RANBP3L (-0.62)***<br>***ZFP36 (-0.62)***<br>***THBS1 (-0.62)***<br>***PLA2G7 (-0.62)***<br>***IGSF6 (-0.61)***<br>***C3AR1 (-0.59)***<br>***CHI3L1 (-0.59)***<br>***MMP9 (-0.59)***<br>***NQO1 (-0.59)***<br>***MS4A6E (-0.59)***<br>***CXorf21 (-0.59)***<br>***MS4A7 (-0.58)***<br>***NPR3 (-0.58)***<br>***MS4A14 (-0.58)***<br>***CCL8 (-0.58)***<br>***P2RY13 (-0.58)***<br>***ITGAX (-0.58)***<br>***JUN (-0.58)***<br>***MS4A4E (-0.57)***<br>***ADAM12 (-0.57)***<br>***MNDA (-0.57)***<br>***CES1 (-0.56)***	***ELOVL6 (12.54)***<br>***SLC27A2 (5.4)***<br>***GYS2 (2.84)***<br>***PKP2 (2.7)***<br>***FASN (2.63)***<br>***ACLY (2.45)***<br>***SCD1 (1.98)***<br>***OLFM2 (1.5)***<br>***LGALS12 (1.46)***<br>***ACACA (1.36)***<br>***THRSP14 (0.78)***<br>***ACSL1 (0.78)***<br>***GLUT4 (0.63)***<br>***DECR1 (0.52)***<br>***ECI1 (0.39)***<br>***IRS-1 (0.38)***	***IL-6 (-0.96)***<br>***FOS (-0.96)***<br>***RGS1 (-0.94)***<br>***AQP9 (-0.90)***<br>***SPP1 (-0.87)***<br>***EGR1 (-0.84)***<br>***DUSP1 (-0.83)***<br>***PRG4 (-0.81)***<br>***EGFL6 (-0.8)***<br>***TNFα (-0.75)***<br>***LYZ (-0.68)***<br>***IQ6GAP2 (-0.51)***<br>***KPNA2 (-0.48)***	(Ortega et al., 2015)

				Upregulated genes involved in:<br><br>(a) Cancer pathways<br>(b) Hematologic disease<br>(c) Post-transcriptional modification of RNA<br>(d) Development of connective tissue<br>(e) Metabolic disease	Note:<br>Downregulated genes involved in:<br>(a) Lipid metabolism<br>(b) Transportation<br>(c) Cell-cell interaction signaling<br>(d) Carbohydrate metabolism<br>(e) Development and function of connective tissue			

Genes, method, N, mean age, BMI (body mass index), reported or calculated fold change, upregulated or downregulated genes, reference. Highlighted genes are those found in the intersection of comparisons between post-RYGB vs obese and lean vs obese. Bold font appoints significance.

Lean vs obese

Method	Characteristics of the groups		Gene expression fold change (Lean vs Obese)		Reference
	Patients data/Recruitment country	BMI (kg/m^2)	Reported fold change [(Lean/Obese)-1]		
			**Upregulated expression in lean (+)**	**Downregulated expression in lean (-)**	
**qPCR**<br>Candidate genes from microarray data Affymetrix GeneChip Human Gene 2.0 ST array >24,838 transcripts between post-surgery vs Obese<br><br>Candidate genes involved in:<br>(a) Cell cycle<br>(b) Development<br>(c) Lipid metabolism<br>(d) Inflammatory response	**Lean:**<br>26 lean of 45±5 years<br><br>**Obese:**<br>25 obese of 48±10 years<br><br>Girona, Spain	**Lean:**<br>~24±2<br><br>**Obese:**<br>~43±5	*3,576 upregulated genes in lean*<br><br>***EGR1 (5.34)***<br>***FOS (2.97)***<br>***PKP2 (2.20)***<br>***ELOVL6 (2.00)***<br>***DUSP1 (1.85)***<br>***GYS2 (1.76)***<br>***SLC27A2 (1.14)***<br>***IL-6 (0.66)***<br>***GLUT4 (0.63)***<br>*RGS1 (0.52)*<br>***DECR1 (0.51)***<br>***OLFM2 (0.51)***<br>***ACSL1 (0.47)***<br>***LGALS12 (0.44)***<br>***FASN (0.43)***<br>***ACLY (0.43)***<br>***THRSP14 (0.43)***<br>***IRS-1 (0.42)***<br>***ECI1 (0.29)***<br>*TNFα (0.18)*<br>*SCD1 (0.12)*<br>*ACACA (0.03)*<br><br>Note: Fold change reported without percentage	*2,420 downregulated genes in lean*<br><br>***LYZ (-0.70)***<br>***SPP1 (-0.70)***<br>***PRG4 (-0.69)***<br>***EGFL6 (-0.64)***<br>***AQP9 (-0.56)***<br>***IQ6GAP2 (-0.52)***<br>***KPNA2 (-0.29)***<br><br>Note: Fold change reported without percentage	(Ortega et al., 2015)

Genes, method, N, mean age, BMI (body mass index), reported or calculated fold change, upregulated or downregulated genes, reference. Highlighted genes are those found in the intersection of comparisons between post-RYGB vs obese and lean vs obese. Bold font appoints significance.

Post-RYGB vs Obese

Method	Characteristics of the groups	Bariatric surgery data	BMI data (kg/m^2)	Gene expression fold change (Post RYGB vs Obese)		Reference
	Patients data/Recruitment country			Reported gene expression	**Calculated fold change [(Post-RYGB/Obese)-1]**	
				**Upregulated expression post-RYGB (+)**		
**Microarrays**<br>Affymetrix Human Gene 1.1 (Affymetrix , Inc., Santa Clara, CA) >28,000 transcripts<br>They focused on associated genes with adipocyte number change in obesity and post-RYGB.	**Obese:** 16 obese of ~46 ± 2 years<br>**Post-RYGB:** 16 patients of ~48 ± 2 years<br>Stockholm, Sweden	RYGB<br>2 years	**Obese:** ~41 ± 1<br>**Post-RYGB:** ~25 ± 1<br>**ΔBMI:** ~16 kg/m^2	*TGFB1*<br>*CXCL2*<br>*FGF2*<br>*OGN*<br>*PDGFD*<br>Upregulated genes involved in growth factors and extracellular matrix organization	Not calculated	(Petrus et al., 2018)

Lean vs Obese

Method	Characteristics of the groups		Gene expression fold change (Lean vs Obese)			Reference
	Patients data/Recruitment country	BMI (kg/m^2)	Reported gene expression		**Calculated fold change [(Lean/Obese)-1]**	
			**Upregulated genes in lean (+)**	**Downregulated genes in lean (-)**		
**Microarrays**<br>Affymetrix Human Gene 1.1 (Affymetrix , Inc., Santa Clara, CA) >28,000 transcripts	**Lean:** 16 lean of ~48 ± 2 years<br>**Obese:** 16 obese of ~46 ± 2 years<br>Stockholm, Sweden	**Lean:** ~25 kg/m^2<br>**Obese**: ~41± 1 kg/m^2	Not reported	*TGFB1*<br>*TGFB3*	Not calculated	(Petrus et al., 2018)

Genes, method, N, mean age, BMI (body mass index), reported or calculated fold change, upregulated or downregulated genes, reference. Highlighted genes are those found in the intersection of comparisons between post-RYGB vs obese and lean vs obese. Bold font appoints significance.

Post-RYGB vs obese

Method	Characteristics of the groups	Bariatric surgery data	BMI data (kg/m^2)	Gene expression fold change (Post RYGB vs Obese)		Reference
	Patients data/Recruitment country			Calculated fold change [(Post-RYGB/Obese)-1]		
				Upregulated expression post-RYGB (+)	Downregulated expression post-RYGB (-)	
				2,420 upregulated genes post-RYGB	*3,576 downregulated genes post-RYGB*	
**Microarrays**<br><br>ClariomTM D arrays 19,418 transcripts excluding non-coding mRNAs<br><br>Focused on inflammatory genes with cutt-of of p-value 10^-8	**Obese:**<br>50 obese of 43±9 years<br><br>**Post-RYGB:**<br>49 patients post-RYGB of 45±9 years<br><br>The surgery candidates<br><br>Stockholm, Sweden	RYGB<br><br>2 years	**Obese:**<br>43±5<br><br>**Post-RYGB:**<br>29±4<br><br>**ΔBMI:**<br>~14 kg/m^2	***PKP2 (1.39)***<br>***C1QB (0.74)***<br>***FASN (0.68)***<br>***SLC4A4 (0.52)***<br>***COX14 (0.45)***<br>***NEDD4L (0.35)***<br>***PRKAR2B (0.32)***<br>***AQP7 (0.31)***<br>***RNF157 (0.31)***<br>***PDE3B (0.3)***<br>***TENM3 (0.27)***	***GPR183 (-0.66)***<br>***LYZ (-0.65)***<br>***CLEC7A (-0.63)***<br>***LCP1 (-0.63)***<br>***NDUFS2 (-0.63)***<br>***VSIG4 (-0.63)***<br>***MS4A7 (-0.62)***<br>***AQP9 (-0.61)***<br>***NCKAP1L (-0.6)***<br>***CTSS (-0.59)***<br>***GLIPR1 (-0.59)***<br>***CYBB (-0.58)***<br>***IGSF6 (-0.58)***<br>***PLEK (-0.58)***<br>***CD163 (-0.57)***<br>***MS4A6A (-0.57)***<br>***TLR8 (-0.57)***<br>***BCAT1 (-0.56)***<br>***TYROBP (-0.56)***<br>***AIF1 (-0.55)***<br>***CD53 (-0.55)***<br>***FCGR2A (-0.55)***<br>***CD68 (-0.54)***<br>***FCGR3A (-0.54)***<br>***TBXAS1 (-0.54)***<br>***CTD-2370N5.3 (-0.53)***<br>***RNASE6 (-0.53)***<br>***CPVL (-0.52)***<br>***F13A1 (-0.51)***<br>***SAMSN1 (-0.51)***<br>***CD4 (-0.5)***<br>***FOLR2 (-0.49)***<br>***IQGAP2 (-0.49)***<br>***PTPRC (-0.49)***<br>***KYNU (-0.48)***<br>***VAMP8 (-0.48)***<br>***RGS10 (-0.47)***<br>***CR1 (-0.46)***<br>***ITGAM (-0.46)***<br>***LYVE1 (-0.45)***<br>***SELPLG (-0.45)***<br>***ADAP2 (-0.44)***<br>***ALOX5AP (-0.44)***<br>***AMICA1 (-0.44)***<br>***ARRB2 (-0.44)***<br>***CSF1R (-0.44)***<br>***ARHGAP30 (-0.43)***<br>***BIN2 (-0.43)***<br>***RPS6KA1 (-0.43)***<br>***SLC7A7 (-0.42)***<br>***GPR137B (-0.4)***<br>***RASSF2 (-0.4)***<br>***CD300A (-0.39)***<br>***SYK (-0.39)***<br>***CD163L1 (-0.38)***<br>***CHST11 (-0.37)***<br>***DOCK10 (-0.37)***<br>***PIK3R5 (-0.37)***<br>***RNASET2 (-0.35)***<br>***CATG00000077766.1 (-0.34)***<br>***CHST15 (-0.33)***<br>***CTSH (-0.33)***<br>***HCLS1 (-0.33)***<br>***PPT1 (-0.33)***<br>***SAMHD1 (-0.33)***<br>***SMAP2 (-0.31)***<br>***SRGN (-0.31)***<br>***PTPLAD2 (-0.3)***	Kerr et al., 2020

Note: Upregulated expression related to protein translation, cell differentiation, and metabolism.<br><br>Note: Some genes with p-value cut-off different than 10^-8 but if p<0.05, were (Calculated fold change at 2 years post-RYGB):<br><br>***SLC2A4 (1.00)***<br>***IRS-1 (0.39)***<br>***ACSL1 (0.15)***<br>***PLIN1 (0.12)***<br>***ADIPOQ (0.09)***	***HGF (-0.29)***<br>***PYCARD (-0.29)***<br>***QPCT (-0.29)***<br>***FUCA1 (-0.27)***<br>***NAIP (-0.27)***<br>***PIK3AP1 (-0.26)***<br>***RNASE1 (-0.25)***<br>***SLC18B1 (-0.25)***<br>***EZR (-0.22)***<br>***CNPY3 (-0.19)***<br><br>Note: downregulated and involved in immune and inflammatory responses.<br><br>Note: Some genes with p-value cut-off different than 10^-8 but if p<0.05, were (Calculated fold change at 2 years post-RYGB):<br><br>***IL-6 (-0.42)***

Genes, method, N, mean age, BMI (body mass index), reported or calculated fold change, upregulated or downregulated genes, reference. Highlighted genes are those found in the intersection of comparisons between post-RYGB vs obese and lean vs obese. Bold font appoints significance.

Post-RYGB vs obese

Method	Characteristics of the groups	Bariatric surgery data	BMI data (kg/m^2)	Gene expression fold change (Post RYGB vs Obese)			Reference
				Calculated fold change [(Post-RYGB/Obese)-1]			
				**Upregulated expression post-RYGB(+)**	**Downregulated expression post-RYGB(+)**		
**<u>Microarrays</u>**<br><br>ClariomTM D arrays<br>19,418 transcripts excluding non-coding mRNAs<br><br>Focused on inflammatory genes with p-value cut-off 10^-8	**Obese:**<br>50 Obese of 43±9 years<br><br>**Post-RYGB:**<br>38 post-RYGB of 47±10 years<br><br>Stockholm, Sweden	RYGB<br><br>5 years	**Obese:**<br>43±5<br><br>**Post-RYGB:**<br>32±6<br><br>**ΔBMI:**<br>~11 kg/m^2	*1,653 upregulated genes post-RYGB*<br><br>Reported fold change<br>**(Post-RYGB/Obese)**<br><br>***PKP2 (2.09)***<br>***FASN (1.14)***<br>***COX14 (0.74)***<br>***NEDD4L (0.56)***<br>***RNF157 (0.51)***<br>***PDE3B (0.43)***<br>***PLEK (0.43)***<br>***TENM3 (0.41)***<br>***PRKAR2B (0.4)***<br>***AQP7 (0.4)***<br><br>Note: Upregulated genes involved in lipid metabolism, adipocyte differentiation and function.<br><br>Note: Some genes with p-value cut-off different than 10^-8 but if p<0.05, were (Calculated fold change at 2 years post-RYGB):<br><br>***SLC2A4 (1.13)***<br>***IRS-1 (0.39)***<br>***ACSL1 (0.20)***<br>***PLIN1 (0.15)***<br>***ADIPOQ (0.09)***	*3,930 downregulated genes post-RYGB*<br><br>Reported fold change<br>**(Post-RYGB/Obese)**<br><br>***AQP9 (-0.77)***<br>***LYZ (-0.78)***<br>***CLEC7A (-0.77)***<br>***LCP1 (-0.76)***<br>***MS4A7 (-0.75)***<br>***NDUFS2 (-0.75)***<br>***VSIG4 (-0.75)***<br>***FCGR3A (-0.72)***<br>***TBXAS1 (-0.72)***<br>***CD53 (-0.72)***<br>***CYBB (-0.72)***<br>***IGSF6 (-0.72)***<br>***CTSS (-0.72)***<br>***TYROBP (-0.71)***<br>***NCKAP1L (-0.71)***<br>***HGF (-0.7)***<br>***HCLS1 (-0.7)***<br>***GPR137B (-0.7)***<br>***CD163 (-0.7)***<br>***GL IPR1 (-0.7)***<br>***GPR183 (-0.7)***<br>***MS4A6A (-0.69)***<br>***TLR8 (-0.69)***<br>***CPVL (-0.68)***<br>***FCGR2A (-0.68)***<br>***SAMSN1 (-0.67)***<br>***CTD-2370N5.3 (-0.67)***<br>***AIF1 (-0.67)***<br>***BCAT1 (-0.66)***<br>***C1QB (-0.65)***<br>***FOLR2 (-0.65)***<br>***F13A1 (-0.65)***<br>***RNASE6 (-0.65)***<br>***CD4 (-0.64)***<br>***CD68 (-0.63)***<br>***AMICA1 (-0.63)***<br>***RGS10 (-0.63)***	***PTPRC (-0.63)***<br>***BIN2 (-0.61)***<br>***ADAP2 (-0.61)***<br>***SELPLG (-0.61)***<br>***LYVE1 (-0.6)***<br>***KYNU (-0.6)***<br>***IQGAP2 (-0.6)***<br>***ALOX5AP (-0.59)***<br>***CSF1R (-0.59)***<br>***CR1 (-0.59)***<br>***VAMP8 (-0.59)***<br>***DOCK10 (-0.57)***<br>***RPS6KA1 (-0.57)***<br>***ITGAM (-0.57)***<br>***ARRB2 (-0.56)***<br>***ARHGAP30 (-0.55)***<br>***SYK (-0.54)***<br>***RASSF2 (-0.54)***<br>***SLC4A4 (-0.53)***<br>***PIK3R5 (-0.51)***<br>***CD300A (-0.51)***<br>***CD163L1 (-0.5)***<br>***CHST11 (-0.49)***<br>***RNASET2 (-0.48)***<br>***SRGN (-0.46)***<br>***CTSH (-0.46)***<br>***CHST15 (-0.45)***<br>***SAMHD1 (-0.45)***<br>***SMAP2 (-0.44)***<br>***SLC7A7 (-0.44)***<br>***PTPLAD2 (-0.43)***<br>***PPT1 (-0.43)***<br>***QPCT (-0.42)***<br>***CATG00000077766.1 (-0.42)***<br>***FUCA1 (-0.4)***<br>***EZR (-0.39)***<br>***PIK3AP1 (-0.38)***<br>***PYCARD (-0.38)***<br>***RNASE1 (-0.36)***<br>***NAIP (-0.34)***<br>***SLC18B1 (-0.33)***<br>***CNPY3 (-0.28)***<br>Note: Downregulated genes involved in immune response and cytokines production<br>Note: Some genes with p-value cut-off different than 10^-8 but if p<0.05, were (Calculated fold change at 2 years post-RYGB):<br>***IL-6 (-0.48)***	(Kerr et al., 2020)

Genes, method, N, mean age, BMI (body mass index), reported or calculated fold change, upregulated or downregulated genes, reference. Highlighted genes are those found in the intersection of comparisons between post-RYGB vs obese and lean vs obese. Bold font appoints significance.

Lean vs obese

Method	Characteristics of the groups		Gene expression fold change (Lean vs Obese)				Reference
	Patients data/Recruitment country	BMI (kg/m^2)	Reported gene expression values **(Obese; Lean)**		**Calculated fold change [(Lean/Obese)-1]**		
			**Downregulated expression in lean (-)**<br>Logarithmic conversion		**Downregulated expression in lean (-)**		
**Microarrays**<br>ClariomTM D arrays >540,000 transcripts<br>Focus on inflammatory genes	**Lean:** 28 lean of 44±9 years<br>**Obese:** 50 obese of 43±9 years<br>Stockholm, Sweden	**Lean:** 27±5<br>**Obese:** 43±5	***LCP1 (584.07;229.13)***<br>***GPR183 (265.03;108.38)***<br>***GLIPR1 (504.95;218.27)***<br>***VSIG4 (364.56;162.02)***<br>***LYZ (1710.26;765.36)***<br>***SAMSN1 (44.94;20.39)***<br>***CD53 (572.05;259.57)***<br>***PLEK (104.69;47.84)***<br>***NCKAP1L (179.77;82.71)***<br>***CTSS (657.11;306.55)***<br>***CLEC7A (113.77;53.08)***<br>***FCGR3A (112.21;53.82)***<br>***CYBB (596.34;290.02)***<br>***MNDA (38.32;19.03)***<br>***TLR8 (34.54;17.39)***<br>***CD68 (1089.92;556.41)***<br>***FCGR2A (1009.9;519.15)***<br>***TYROBP (1686.71;879.17)***<br>***FCGR3B (108.38;57.68)***<br>***AIF1 (1168.14;625.99)***<br>***IQGAP2 (126.24;67.65)***<br>***BIN2 (89.88;49.52)***<br>***VAMP8 (100.43;56.1)***<br>***CR1 (39.4;22.78)***<br>***PTPRC (439.59;254.23)***<br>***PRKCB (61.82;36.5)***<br>***RPS6KA1 (45.89;27.1)***<br>***FOLR2 (265.03;157.59)***<br>***SYK (40.5;24.42)***<br>***ITGAM (121.1;73.01)***<br>***FPR1 (63.56;38.59)***<br>***CXCR4 (276.28;167.73)***<br>***LY86 (99.73;61.39)***<br>***CD4 (148.06;91.14)***<br>***C1QA (430.54;265.03)***<br>***ALOX5AP (568.1;352.14)***<br>***ARRB2 (634.73;398.93)***<br>***MYO1F (245.57;158.68)***<br>***RNASET2 (157.59;103.97)***	***SAMHD1 (685.02;458.25)***<br>***LGMN (1031.12;704.28)***<br>***HGF (91.14;62.68)***<br>***CORO1A (200.85;138.14)***<br>***S100A8 (127.12;88.65)***<br>***LY96 (17.15;11.96)***<br>***CD48 (41.93;29.65)***<br>***FCER1A (41.36;29.24)***<br>***FUCA1 (173.65;127.12)***<br>***QPCT (37.79;28.05)***<br>***NPC2 (694.58;519.15)***<br>***PIK3AP1 (133.44;101.83)***<br>***SELL (53.45;41.64)***<br>***EZR (324.03;261.38)***<br>***CTSC (861.08;694.58)***<br>***CNPY3 (166.57;138.14)***<br>***GMFG (861.08;714.11)***<br>***TNFRSF1B (418.77;362.04)***<br>***ZC3HAV1 (221.32;198.09)***<br>***FGL2 (458.25;424.61)***<br>***CYLD (123.64;117.78)***	***LCP1 (-0.61)***<br>***GPR183 (-0.59)***<br>***GLIPR1 (-0.57)***<br>***VSIG4 (-0.56)***<br>***LYZ (-0.55)***<br>***SAMSN1 (-0.55)***<br>***CD53 (-0.55)***<br>***PLEK (-0.54)***<br>***NCKAP1L (-0.54)***<br>***CTSS (-0.53)***<br>***CLEC7A (-0.53)***<br>***FCGR3A (-0.52)***<br>***CYBB (-0.51)***<br>***MNDA (-0.5)***<br>***TLR8 (-0.5)***<br>***CD68 (-0.49)***<br>***FCGR2A (-0.49)***<br>***TYROBP (-0.48)***<br>***FCGR3B (-0.47)***<br>***AIF1 (-0.46)***<br>***IQGAP2 (-0.46)***<br>***BIN2 (-0.45)***<br>***VAMP8 (-0.44)***<br>***CR1 (-0.42)***<br>***PTPRC (-0.42)***<br>***PRKCB (-0.41)***<br>***RPS6KA1 (-0.41)***<br>***FOLR2 (-0.41)***<br>***SYK (-0.4)***<br>***ITGAM (-0.4)***<br>***FPR1 (-0.39)***<br>***CXCR4 (-0.39)***<br>***LY86 (-0.38)***<br>***CD4 (-0.38)***<br>***C1QA (-0.38)***<br>***ALOX5AP (-0.38)***<br>***ARRB2 (-0.37)***<br>***MYO1F (-0.35)***<br>***RNASET2 (-0.34)***	***SAMHD1 (-0.33)***<br>***LGMN (-0.32)***<br>***HGF (-0.31)***<br>***CORO1A (-0.31)***<br>***S100A8 (-0.3)***<br>***LY96 (-0.3)***<br>***CD48 (-0.29)***<br>***FCER1A (-0.29)***<br>***FUCA1 (-0.27)***<br>***QPCT (-0.26)***<br>***NPC2 (-0.25)***<br>***PIK3AP1 (-0.24)***<br>***SELL (-0.22)***<br>***EZR (-0.19)***<br>***CTSC (-0.19)***<br>***CNPY3 (-0.17)***<br>***GMFG (-0.17)***<br>***TNFRSF1B (-0.14)***<br>***ZC3HAV1 (-0.1)***<br>***FGL2 (-0.07)***<br>***CYLD (-0.05)***	(Kerr et al.,2020)

Genes, method, N, mean age, BMI (body mass index), reported or calculated fold change, upregulated or downregulated genes, reference. Highlighted genes are those found in the intersection of comparisons between post-RYGB vs obese and lean vs obese. Bold font appoints significance.

Lean vs obese

Method	Characteristics of the groups		Gene expression fold change (Lean vs Obese)				Reference
	Patients data/Recruitment country	BMI (kg/m^2)	Reported gene expression values **(Obese; Lean)**		**Calculated fold change [(Lean/Obese)-1]**		
			**Upregulated expression in lean (+)**	**Downregulated expression in lean (-)**	**Upregulated expression in lean**	**Downregulated expression in lean (-)**	
**qPCR**<br><br>Candidate genes involved in:<br>(a) Adipogenic markers<br>(b) Insulin signaling pathway<br>(c) Remodeling of the extracellular matrix (ECM)<br>(d) Inflammatory	**Lean:**<br>83 lean<br>of ~23±2 years<br><br>**Obese:**<br>19 obese<br>of ~26±5 years<br><br>Bialystok, Poland	**Lean:**<br>~22 ± 2<br><br>**Obese:**<br>~33 ± 3	***SLC2A4 (0.20; 0.49)***<br>*MAPK11 (0.04; 0.08)*<br>*TLR2 (0.01; 0.02)*<br>***CEBPB (0.10; 0.19)***<br>***IRS-2 (0.58; 0.9)***<br>***NFKBIA (0.17; 0.25)***<br>*PIK3CA (0.13; 0.19)*<br>*MAPK14 (0.56; 0.8)*<br>***ADIPOQ (0.48; 0.68)***<br>***PPARG (0.95; 1.34)***<br>***VEGFA (0.29; 0.41)***<br>***CEBPA (4.05; 5.69)***<br>*TLR4 (0.14; 0.19)*<br>***IRS-1 (0.59; 0.78)***<br>*NFKBIL1 (0.18; 0.23)*<br>*MMP2 (0.38; 0.48)*<br>***AKT2 (2.32; 2.9)***<br>*JUN (0.34; 0.42)*<br>*COL1A1 (0.78; 0.95)*<br>*MAPK8 (0.1; 0.12)*<br>*RELA (0.45; 0.5)*<br>*CCL3 (0.10; 0.11)*<br>*RASA1 (0.34; 0.37)*<br>*MAP3K1 (0.13; 0.14)*<br>*GRB2 (0.57; 0.6)*<br>*SREBF1 (0.28; 0.29)*<br>*MAPK1 (0.31; 0.32)*<br>*RAF1 (0.026; 0.028*<br>*CD8A (0.010; 0.013)*<br><br>Note: Approximate values from the graphs in the article	***IL-18 (0.03; 0.01)***<br>***MMP9 (0.26; 0.09)***<br>***CCL2 (0.1; 0.05)***<br>***CD14 (0.64; 0.35)***<br>***ITGAM (0.07; 0.04)***<br>***CD68 (0.31; 0.18)***<br>***ITGAX (0.11; 0.07)***<br>*CD19 (0.03; 0.02)*<br>***CD86 (0.03; 0.02)***<br>*NFKB2 (0.12; 0.09)*<br>***IKBKB (0.04; 0.03)***<br>***TIMP1 (0.34; 0.27)***<br>*MIF (0.36; 0.31)*<br>*CD40 (0.07; 0.06)*<br>*NFKB1 (0.08; 0.07)*<br>*CD4 (0.042; 0.040)*<br>*CD3D (0.02; 0.01)*<br>*FOXP3 (0.009; 0.007)*<br><br>Note: Approximate values from the graphs in the article	***SLC2A4 (1.45)***<br>*MAPK11 (1.00)*<br>*TLR2 (1.00)*<br>***CEBPB (0.90)***<br>***IRS-2 (0.55)***<br>***NFKBIA (0.47)***<br>*PIK3CA (0.46)*<br>*MAPK14 (0.43)*<br>***ADIPOQ (0.42)***<br>***PPARG (0.41)***<br>***VEGFA (0.41)***<br>***CEBPA (0.40)***<br>*TLR4 (0.36)*<br>***IRS-1 (0.32)***<br>*NFKBIL1 (0.28)*<br>*MMP2 (0.26)*<br>***AKT2 (0.25)***<br>*JUN (0.24)*<br>*COL1A1 (0.22)*<br>*MAPK8 (0.2)*<br>*RELA (0.11)*<br>*CCL3 (0.10)*<br>*RASA1 (0.09)*<br>*MAP3K1 (0.08)*<br>*GRB2 (0.05)*<br>*SREBF1 (0.04)*<br>*MAPK1 (0.03)*<br>*RAF1 (0.08)*<br>*CD8A (0.30)*	***IL-18 (-0.67)***<br>***MMP9 (-0.65)***<br>***CCL2 (-0.50)***<br>***CD14 (-0.45)***<br>***ITGAM (-0.43)***<br>***CD68 (-0.42)***<br>***ITGAX (-0.36)***<br>*CD19 (-0.33)*<br>***CD86 (-0.33)***<br>*NFKB2 (-0.25)*<br>***IKBKB (-0.25)***<br>***TIMP1 (-0.21)***<br>*MIF (-0.14)*<br>*CD40 (-0.14)*<br>*NFKB1 (-0.13)*<br>*CD4 (-0.05)*<br>*CD3D (-0.15)*<br>*FOXP3 (-0.22)*	(Matulewicz et al., 2017)

Genes, method, N, mean age, BMI (body mass index), reported or calculated fold change, upregulated or downregulated genes, reference. Highlighted genes are those found in the intersection of comparisons between post-RYGB vs obese and lean vs obese. Bold font appoints significance.

Lean vs obese

Method	Characteristics of the groups		Gene expression fold change (Lean vs Obese)				Reference
	Patients data/Recruitment country	BMI (kg/m^2)	Microarrays: Upregulated expression in lean (+)	Microarrays: Downregulated expression in lean (-)	qPCR: Reported gene expression values (Obese; Lean)	qPCR: Calculated fold change [(Lean/Obese)-1]	
**Microarrays**<br>Two-Color Microarray Based Gene Expression Analysis/Low Input Quick Amp Labeling >18,000 transcripts<br>**qPCR**<br>qPCR validation of only 3 genes selected by the author<br>Note: In both cases, the selection criteria are not explained, only that they are involved in obesity.	**Microarrays**<br>**Lean:**<br>8 lean of ~36±9 years<br>**Obese:**<br>8 obese of ~41±9 years<br>**qPCR**<br>**Lean:**<br>35 patients<br>**Obese:**<br>37 obese<br>Mexico City, Mexico	**Microarrays**<br>**Lean:**<br>~24±1<br>**Obese:**<br>~33±3	***ADPN, PPARG, IRS-2, CEBPB, MEF2A, MEF2D, MVD, ACSL1, ACSL3, GAPDHS, PGK1, LDHC, IRS, MAP3K2, MAP3K6, MAP3K9, MAP3K14, AKT1, ABCD1, CDKN2A, CASP4, CASP11, PNPLA3.***<br>Note: They do not present numeric expression values, only that they are upregulated.<br>Genes involved in:<br>(a) Endocrine function of the adipocyte<br>(b) Cholesterol and triglyceride synthesis<br>(c) Beta-oxidation of fatty acids<br>(d) Insulin signaling<br>(e) Leptin signaling<br>(f) Cytochromes p450	***RETN, PPARA, SREBP2, HKI, CD40LG, VTN, ALOX5, RAPGEF1, BAX, BAX, CYP7A1, CYP19A1***<br>Note: They do not present numeric expression values, only that they are overexpressed.<br>Genes involved in:<br>(a) Glycolysis and gluconeogenesis<br>(b) Inflammation<br>(c) Insulin signaling<br>(d) Leptin signaling<br>(e) TG synthesis<br>(f) Carbohydrate metabolism<br>(g) Oxidative stress<br>(h) Cytochromes p450	**Upregulated expression in lean (+)**<br>*MVD (0.37; 1.00)*<br>***ADPN (0.59; 1.00)***<br>***PPARG (0.90; 1.00)***<br>Note: Approximate values from graphics in the article	**Upregulated expression in lean (-)**<br>*MVD (1.70)*<br>***ADPN (0.69)***<br>***PPARG (0.11)***	(Ronquillo et al., 2019)

Genes, method, N, mean age, BMI (body mass index), reported or calculated fold change, upregulated or downregulated genes, reference. Highlighted genes are those found in the intersection of comparisons between post-RYGB vs obese and lean vs obese. Bold font appoints significance.

Lean vs obese

Method	Characteristics of the groups		Gene expression fold change (Lean vs Obese)		Reference
	Patients data/Recruitment country	BMI (kg/m^2)	Reported gene expression		
			**Upregulated genes in lean (+)**	**Downregulated genes in lean (-)**	
**Microarrays**<br>Affymetrix Human Gene 2.1ST array<br>10,000 transcripts<br><br>Genes involved in:<br>(a) Cytokine-cytokine receptor interaction<br>(b) Chemokine signaling pathway	**Lean:**<br>9 lean of 50 ± 3 years<br><br>**Obese:**<br>11 Obese of 46 ± 1 years<br><br>Ontario, Canada	**Lean:**<br>~22 ± 2<br><br>**Obese:**<br>~35 ± 1	91 upregulated genes in lean<br><br>*PPP1R1B*<br>*NPR1*<br>*HCAR3*<br>*P1K3CA*<br><br>Note:<br>Significantly upregulated genes involved in:<br>(a) Lipolysis in adipocytes<br>(b) cAMP signaling pathway	130 downregulated genes in lean<br><br>*CCL19*<br>*CXCL10*<br>*CXCL11*<br>*TNFRSF11A*<br>*TNFRSF25*<br>*PDGFA*<br>*GNB4*<br>*ADCY1*<br><br>Note:<br>Significantly downregulated genes involved in:<br>(a) Cytokine-cytokine receptor interaction<br>(b) Chemokine signaling pathway	(Badoud et al., 2017)

Genes, method, N, mean age, BMI (body mass index), reported or calculated fold change, upregulated or downregulated genes, reference. Highlighted genes are those found in the intersection of comparisons between post-RYGB vs obese and lean vs obese. Bold font appoints significance.
