# Supplementary figures and images for: Differential Gene Expression of Subcutaneous Adipose Tissue among Lean, Obese, and after RYGB (Different Timepoints): Systematic Review and Analysis
Source: Nutrients. 2022 Nov 21;14(22):4925. doi: 10.3390/nu14224925 (PMC9693162; doi:10.3390/nu14224925)

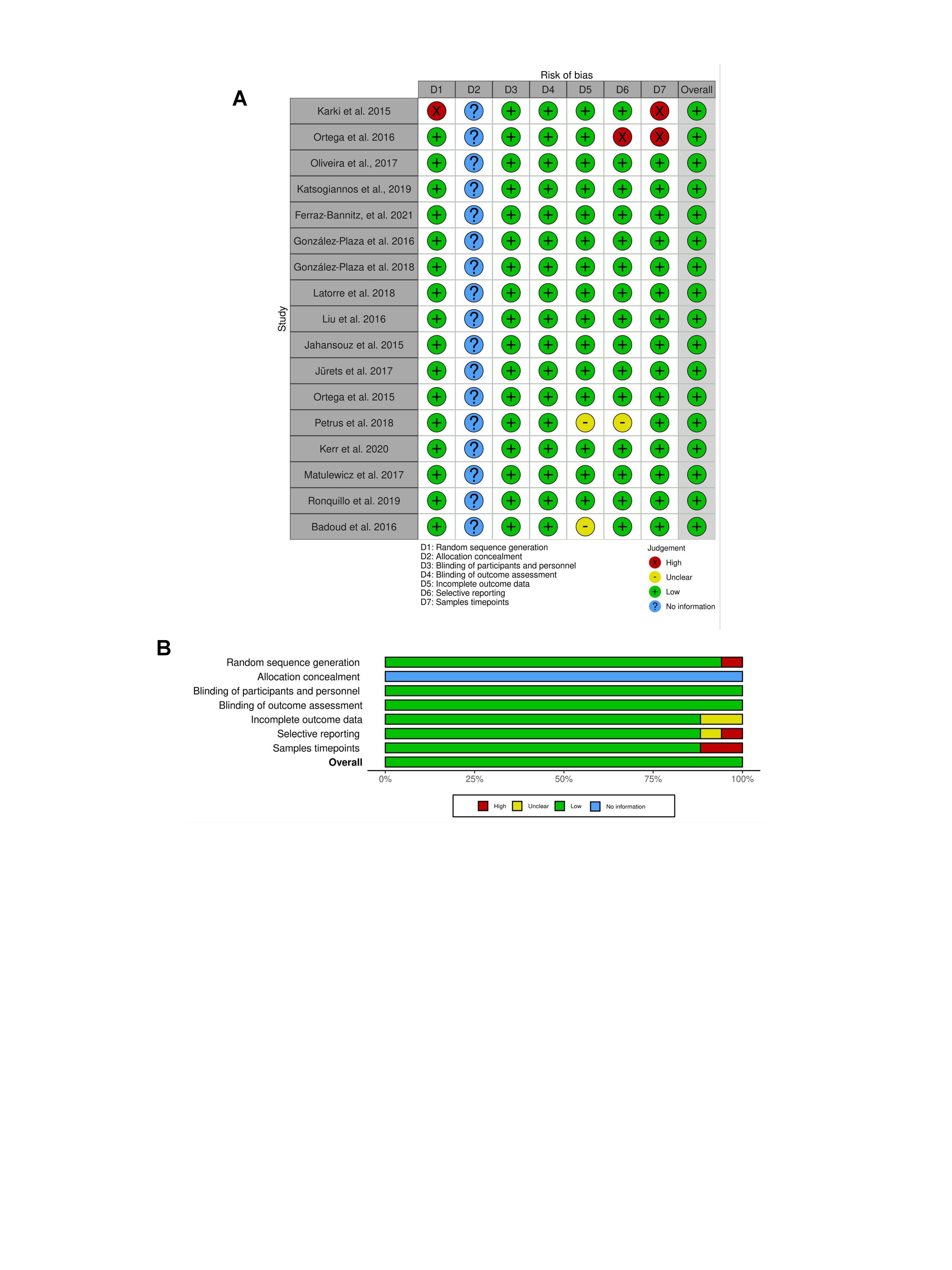

Supplement: Supplementary file 1 [file nutrients-14-04925-s001.zip › Figure S1. Risk of bias graph.jpg]
